# Review of the distribution of Kaposi's sarcoma-associated herpesvirus (KSHV) in Africa in relation to the incidence of Kaposi's sarcoma

**DOI:** 10.1038/sj.bjc.6600745

**Published:** 2003-01-28

**Authors:** M Dedicoat, R Newton

**Affiliations:** ^1^Liverpool School of Tropical Medicine, Hlabisa Hospital and the Africa Centre for Population Studies and Reproductive Health, PO Box 252, Hlabisa 3937, KwaZulu/Natal, South Africa; ^2^Cancer Research UK, Epidemiology Unit, Gibson Building, Radcliffe Infirmary, Oxford OX2 6HE, UK

**Keywords:** KSHV/HHV-8, Kaposi's sarcoma, Africa

## Abstract

In the years before human immunodeficiency virus (HIV) infection, the incidence of Kaposi's sarcoma varied markedly across the African continent, and it was a disease primarily affecting men. In contrast, the evidence reviewed here shows that the causal virus–Kaposi's sarcoma associated herpesvirus (KSHV)–is prevalent in many African countries, including places where Kaposi's sarcoma was almost unknown before HIV, and that it is as common in women as in men. Therefore, the geographical distribution of Kaposi's sarcoma in Africa before the spread of HIV and its predominance as a disease affecting men are not a simple reflection of the distribution of KSHV. Since the epidemic of HIV in Africa, Kaposi's sarcoma has become relatively more frequent in women, and the incidence has increased in countries where it was previously rare, but where KSHV is prevalent, as well as in countries where it was already common. These changes point to a role for other (as yet unknown) factors in the aetiology of Kaposi's sarcoma that may have the most effect in the absence of concurrent HIV infection.

Before the human immunodeficiency virus (HIV) epidemic, Kaposi's sarcoma had a much greater geographical variation in incidence than most other malignancies. It was as common in parts of sub-Saharan Africa, such as Uganda, as colon cancer is in Europe and the USA, representing up to 9% of all cancers in men (Oettlé, 1962; D'Oliveira and Torres, 1972; Templeton, 1981; Hutt, 1984; Cook-Mozaffari *et al*, 1998). Kaposi's sarcoma was also endemic, although much rarer, in counties around the Mediterranean, particularly in Italy, Greece and the Middle East, but was almost nonexistent elsewhere, except in immigrants from these endemic countries (Biggar *et al*, 1984; Grulich *et al*, 1992; Hjalgrim *et al*, 1996). The pattern of geographical variation in incidence correlates broadly with the worldwide distribution of Kaposi's sarcoma-associated herpesvirus (KSHV; human herpesvirus-8 (HHV-8)), which is considered to be a necessary cause of Kaposi's sarcoma (Chang *et al*, 1994,^1996^; Gao *et al*, 1996; Simpson *et al*; 1996; Boshoff, 1999). Here, we review published data on the prevalence of infection with KSHV across Africa and make comparison with the distribution of Kaposi's sarcoma before and since the start of the HIV epidemic.

## Methods

Studies published up to the end of March 2002 that looked for evidence of infection with KSHV among people living in Africa were identified through a Medline search, supplemented by an examination of references given in identified papers and by searching the index of relevant journals. No restriction was placed on the language of publication, and foreign language papers were translated. Only peer-reviewed journals were considered and no attempt was made to identify unpublished studies or to obtain unpublished data from published studies. Conference abstracts, which are often based on preliminary analyses, were excluded. We adopted the criteria that had been used within individual studies for evidence of KSHV infection. Many of the identified studies supplied prevalence estimates for children and adults, or estimates derived from the use of more than one KSHV assay–the full range of available estimates are shown in Table 1

**Table 1:** Reported prevalence of evidence of infection with KSHV in Africa, in relation to the cumulative incidence of Kaposi's sarcoma in men in the years before the HIV epidemic

**African country**	**Prevalence of evidence of KSHV infection (%)**	**Cumulative incidence of KS (per 1000) in men, before 1980^a^**
Uganda	14–86	9
Cameroon	28–62	8
Democratic Republic of Congo	25–82	7
Tanzania	66	6
Zambia	8–58	3
Nigeria	6–56	3
Zimbabwe	5–32	3
Ivory Coast	43–100	3
South Africa	16–75	2
Botswana	76–87	1
Ghana	34–43	1
The Gambia	29–84	0
Egypt	7–45	0
Senegal	14	No data
Central African Republic	23	No data
Eritrea	2–26	No data

a Data on the cumulative incidence of Kaposi's sarcoma in Africa were extracted from Cook−Mozaffari *et al* (1998). It is not known if data on KSHV from the same patient samples were published in more than one study.

References for each country: (percentage of those tested who were KSHV positive for each study) and (type of detection test used).

*Uganda*–Chang *et al* (1996) (14%) (DNA detection); Gao *et al* (1996) (51–71%) (latent IFA); Lennette *et al* (1996) (11–77%) (lytic and latent IFA); Simpson *et al* (1996) (35–53%) (latent IFA, ORF65 ELISA); Mayama *et al* (1998) (37–60%) (latent IFA, ORF65 ELISA); de Thé *et al* (1999) (86%) (lytic and latent IFA); Ablashi *et al* (1999) (39–46%) (whole cell ELISA); Kakoola *et al* (2001) (74%) (ELISA ORF65, 73 and latent IFA); Serraino *et al* (2001) (26–47%) (lytic and latent IFA); Wawer *et al* (2001) (36–45%) (latent IFA, ORF65 ELISA). *Cameroon*–Bestetti *et al* (1998) (38–57%) (latent IFA); Gessain *et al* (1999) (28–48%) (lytic and latent IFA); Rezza *et al* (2000) (40–62%) (lytic and latent IFA); Serraino *et al* (2001) (47–53%) (lytic and latent IFA). *Democratic Republic of Congo*–Lennette *et al* (1996) (25–82%) (lytic and latent IFA); Engels *et al* (2000) (82%) (latent IFA, ELISA ORF65, K8.1). *Tanzania*–de Thé *et al* (1998) (66%) (lytic and latent IFA). *Zambia*–Kasolo *et al* (1997) (8%) (DNA detection); He *et al* (1998) (48%) (lytic IFA); Olsen *et al* (1998) (58%) (latent IFA, ORF65 WB); Ablashi *et al* (1999) (9–44%) (whole cell ELISA). *Nigeria*–Lennette *et al* (1996) (6–56%) (lytic and latent IFA). *Zimbabwe*–Lennette *et al* (1996) (11–32%) (lytic and latent IFA); Lampinen *et al* (2000) (5%) (ORF65 ELISA). *Ivory Coast*–Lennette *et al* (1996) (43–100%) (lytic and latent IFA). *South Africa*–Bourboulia *et al* (1998) (16%) (latent IFA); Sitas *et al* (1999) (32%) (latent IFA); Wilkinson *et al* (1999) (38–75%) (latent IFA, ORF65 ELISA). *Botswana*–Engels *et al* (2000) (76–87%) (latent IFA, ELISA ORF65, K8.1). *Ghana*–Ablashi *et al* (1999) (42%) (whole cell ELISA); Nuvor *et al* (2001) (34–43%) (ELISA ORF59,65,73, K8.1). The *Gambia*–Lennette *et al* (1996) (29–84%) (lytic and latent IFA); Ariyoshi *et al* (1998) (63–83%) (latent IFA, ORF65 ELISA). *Egypt* – Andreoni *et al* (1999) (7–45%) (lytic and latent IFA); Serraino *et al* (2001) (42–43%) (lytic and latent IFA); Andreoni *et al* (2002) (42%) (lytic and latent IFA). *Senegal*–Gaye−Diallo *et al* (2001) (14%) (lytic and latent IFA). *Central African Republic*–Belec *et al* (1998) (23%) (DNA detection). *Eritrea*–Enbom *et al* (1999) (5–26%) (lytic and latent IFA).

. Cumulative incidence (for ages 0–64) of Kaposi's sarcoma in men, prior to 1980, was obtained from a published report by Cook-Mozaffari *et al* (1998). Where regional rates within a given country were available, they were used to calculate a country-wide mean incidence rate. Brief details of the assays used in each study are included in the footnote to the table, but this report is not intended as a review of methods of detection of KSHV infection.

## Results and Discussion

In all, we identified 28 studies that had measured the prevalence of KSHV infection in 16 African countries. Of these, three studies identified evidence of KSHV-DNA in peripheral blood cells (Chang *et al*, 1996; Kasolo *et al*, 1997; Belec *et al*, 1998), two used a lytic antibody assay alone (He *et al*, 1998; Lampinen *et al*, 2000), five used a latent antibody assay alone (Gao *et al*, 1996; Ariyoshi *et al*, 1998; Bestetti *et al*, 1998; Bourboulia *et al*, 1998; Sitas *et al*, 1999) and 18 used both a lytic and a latent assay (Lennette *et al*, 1996; Simpson *et al*, 1996; Mayama *et al*, 1998; Olsen *et al*, 1988; de Thé *et al*, 1999; Ablashi *et al*, 1999; Andreoni *et al*, 1999,^2002^; Enbom *et al*, 1999; Gessain *et al*, 1999; Wilkinson *et al*, 1999; Engels *et al*, 2000; Rezza *et al*, 2000; Gaye Serraino-Diallo *et al*, 2001; Kakoola *et al*, 2001; Nuvor *et al*, 2001; Serraino *et al*, 2001; Wawer *et al*, 2001). The results are presented in Table1, together with the estimated cumulative incidence of Kaposi's sarcoma in men aged 0–64 years in the period before HIV infection (Cook-Mozaffari *et al*, 1998).

KSHV is common in countries such as Uganda and Cameroon, where Kaposi's sarcoma was relatively frequent, but the virus is also common in countries such as Botswana and the Gambia, where Kaposi's sarcoma rarely occurred before the spread of HIV infection. There was no evidence in any of the studies that the prevalence of KSHV (however it was measured) differed between men and women. Before the onset of the HIV epidemic in the 1980s, Kaposi's sarcoma showed extreme geographical variation in incidence even within the African continent. Narrow belts of relatively high incidence stretched westward across the former Zaire to the coast of Cameroon and southward down the rift valley to Malawi. In all of these areas, Kaposi's sarcoma was more common in men than in women (Cook-Mozaffari *et al*, 1998).

There is no evidence that the prevalence of KSHV has changed in Africa since the spread of HIV. Indeed, KSHV, a *γ*2 herpesvirus, is thought to have coexisted alongside *Homo sapiens* since their origin (reviewed by Hayward, 1999). Furthermore, the study by de Thé *et al* (1999) has shown that anti-KSHV antibodies were present in Africa prior to the HIV epidemic at levels similar to those seen today. Why then, was Kaposi's sarcoma not more common in African countries that have a high prevalence of KSHV and why, when the virus is equally prevalent in men as it is in women, was Kaposi's sarcoma so much more frequent in men? Both anomalies suggest the importance of further (as yet unknown) cofactors in the aetiology of the tumour.

Parts of Africa with a high prevalence of HIV and where Kaposi's sarcoma was relatively common even in the years before acquired immunodeficiency syndrome (AIDS) have seen an explosion in the incidence of the tumour. In the last 10–15 years, the incidence of Kaposi's sarcoma has increased about 20-fold in Uganda and Zimbabwe, such that it is now the most common cancer in men and the second most common in women (Wabinga *et al*, 1993,^2000^; Bassett *et al*, 1995). As a result of the HIV epidemic, the incidence of Kaposi's sarcoma has also increased in countries where it was previously relatively rare, but where KSHV was prevalent. For example, between 1988 and 1996, the incidence of Kaposi's sarcoma has risen at least three-fold in South Africa and continues to increase as the HIV epidemic grows. Data from Johannesburg, South Africa, show that incidence rates of Kaposi's sarcoma have doubled in men, but have increased seven-fold in women, such that the sex ratio of 7 : 1 in males *vs* females in 1988 has now declined to only 2 : 1 (Sitas and Newton, 2000). The role of other cofactors in the aetiology of Kaposi's sarcoma may, therefore, be less relevant in the presence of HIV infection than they seem to have been for the development of classical Kaposi's sarcoma prior to the spread of HIV.
